# In-Situ Construction of Fe-Doped NiOOH on the 3D Ni(OH)_2_ Hierarchical Nanosheet Array for Efficient Electrocatalytic Oxygen Evolution Reaction

**DOI:** 10.3390/ma17184670

**Published:** 2024-09-23

**Authors:** Mengyang Li, Mingran Wang, Qianwei Wang, Yang Cao, Jie Gao, Zhicheng Wang, Meiqi Gao, Guosheng Duan, Feng Cao

**Affiliations:** 1 Key Lab for Anisotropy and Texture of Materials (MoE), School of Materials Science and Engineering, Northeastern University, Shenyang 110819, China; 2310170@stu.neu.edu.cn (M.L.); 2200639@stu.neu.edu.cn (M.W.); 2270554@stu.neu.edu.cn (Q.W.); 2270359@stu.neu.edu.cn (Y.C.); 2300569@stu.neu.edu.cn (J.G.); 2300688@stu.neu.edu.cn (Z.W.); gaomq@smm.neu.edu.cn (M.G.); 2 School of Safety Engineering, Shenyang Aerospace University, Shenyang 110136, China

**Keywords:** OER activity, Fe-doped NiOOH, heterostructure, water-splitting, electrocatalysts

## Abstract

Accessible and superior electrocatalysts to overcome the sluggish oxygen evolution reaction (OER) are pivotal for sustainable and low-cost hydrogen production through electrocatalytic water splitting. The iron and nickel oxohydroxide complexes are regarded as the most promising OER electrocatalyst attributed to their inexpensive costs, easy preparation, and robust stability. In particular, the Fe-doped NiOOH is widely deemed to be superior constituents for OER in an alkaline environment. However, the facile construction of robust Fe-doped NiOOH electrocatalysts is still a great challenge. Herein, we report the facile construction of Fe-doped NiOOH on Ni(OH)_2_ hierarchical nanosheet arrays grown on nickel foam (FeNi@NiA) as efficient OER electrocatalysts through a facile in-situ electrochemical activation of FeNi-based Prussian blue analogues (PBA) derived from Ni(OH)_2_. The resultant FeNi@NiA heterostructure shows high intrinsic activity for OER due to the modulation of the overall electronic energy state and the electrical conductivity. Importantly, the electrochemical measurement revealed that FeNi@NiA exhibits a low overpotential of 240 mV at 10 mA/cm^2^ with a small Tafel slope of 62 mV dec^−1^ in 1.0 M KOH, outperforming the commercial RuO_2_ electrocatalysts for OER.

## 1. Introduction

The electrochemical decomposition of water is of considerable importance for the conversion and storage of renewable fuel cells and substitutable energy sources such as hydrogen, overcoming the difficulties of the depletion of non-renewable energy and serious environmental pollution [1,2,3]. Nonetheless, the oxygen evolution reaction (OER), an important half-cell reaction during water splitting, is severely restricted by its sluggish kinetic process [2,4]. Consequently, it is imperative to develop effective electrocatalysts that can expedite the reaction process and improve overall efficiency. Rare metal-derived materials, such as RuO_2_ and IrO_2_, are deemed the cutting-edge electrocatalysts for OER [5]. Despite their benefits, their industrial implementation is obstructed by the disadvantages of rarity, expensiveness, and deficient durability [6,7]. Hence, much effort has been put into the exploration of earth-abundant transition metal elements for developing catalysts with superior activity and endurance.

Transition metal-based oxides and hydroxides are extensively investigated as OER electrocatalysts attributed to their economic viability, high abundance, and unique 3D electronic structures [8,9]. Notably, Fe- and Ni-based hydroxides are widely deemed to be identified as the foremost option for OER catalysis in alkaline media. For instance, various Fe/Ni-based oxides and hydroxides with distinct morphological, structural, and compositional attributes have been developed to improve OER activities [10,11,12]. Among them, the corresponding oxyhydroxides produced from surface reconstruction during OER were deemed to be one of the most active species under alkaline conditions [13,14,15,16,17,18,19,20,21,22,23]. Moreover, the optimized electronic structure and coordination sphere around Ni atoms by Fe-doping can expedite the rate of electron transfer and decrease the kinetic barrier. Hence, the Fe-doping can further enhance the intrinsic activity of Ni (oxy)hydroxides by about 30 and 1000 times [15,19,24,25,26,27,28].

Ni foam can be used as a substrate to -fabricate Ni(OH)_2_ hierarchical structures in situ for catalysis applications as a result of its excellent electrical conductivity and high porosity [29,30,31,32,33,34,35]. For instance, Kou et al. presented an accessible hydrothermal technique to modify Ni foam by applying Fe-doped β-Ni(OH)_2_ nanosheets for boosting OER performance [36]. Nevertheless, a universal and fast approach to construct Fe-doped NiOOH for the efficient controlling of the surface reconstruction to achieve enhanced electrocatalysis for OER is rarely reported [37,38,39,40]. Previous studies have shown that FeNi PBA with a high surface area and uniform porosity can be reconstructed as Fe-doped NiOOH for enhanced oxygen evolution catalysis by cyclic voltammetry conditioning [41,42]. Nevertheless, PBA-derived catalysts exhibit low electrocatalytic activity and stability because of the poor conductivity of powder and mechanical loss [43,44,45]. Thus, a deeper in situ electrochemical activation to form Fe-doped NiOOH on 3D FeNi PBA as an open framework would be expected to be as effective and robust OER electrocatalysts.

In this work, FeNi-based PBA nanocubes were prepared via treating Ni(OH)_2_ nanoarrays established on nickel foam through a mild ion exchange reaction. The OER electrocatalytic properties of Fe-doped NiOOH@Ni(OH)_2_ were further enhanced by the electrochemical activating of the FeNi-PBA@Ni(OH)_2_/Ni precursor. The incorporation of Fe is expected to modify the electronic structures and coordination environment of active atoms, promote the formation of Fe-doped NiOOH@Ni(OH)_2_ heterostructures, and then improve the electron transfer rate by decreasing the kinetic barrier, thereby enhancing the OER performance. This well-designed composite exhibits a prominent performance for OER with a minimal overpotential of 240 mV at the current density of 10 mA/cm^2^ and an ultralow Tafel slope of 62 mV dec^−1^, which outperform recently reported catalysts, even RuO_2_. This work adopts an electrochemical activation strategy, which proposes a new approach to the mechanism of reconstructing PBA or PBA derivatives on metal hydroxide substrates to synthesize metal-doped (oxy)hydroxide heterostructures.

## 2. Materials and Methods

### 2.1. Materials

The reagents Nickel nitrate hexahydrate (Ni(NO_3_)_2_·6H_2_O), potassium ferricyanide (K_3_[Fe(CN)_6_]), urea, ammonium fluoride (NH_4_F), nickel foam (NF), ruthenium dioxide (RuO_2_), and Nafion (5 wt%) were all purchased from Sinopharm Group.

### 2.2. Preparation of Ni(OH)_2_/Ni

Ni(NO_3_)_2_·6H_2_O (0.131 g), urea (0.225 g), and NH_4_F (0.056 g) were mixed in 150 mL of water and stirred with a magnetic stirrer until fully dissolved. The size of Ni foam was 1.5 cm × 4 cm with a thickness of 1.5 mm. It was cleaned by acetone and ethanol under ultrasonication conditions for 10 min, respectively, and then rinsed repeatedly with ethanol and water. The nickel foam was placed into the solution in a 100 mL Teflon-lined autoclave and heated at 120 °C in a drying oven for 2 h. The prepared sample was named Ni(OH)_2_/NF.

### 2.3. Preparation of FeNi-PBA@Ni(OH)_2_/Ni

Add 0.033 g of K_3_[Fe(CN)_6_] to 20 mL of water and stir magnetically until the substance is completely dissolved. The nickel foam with Ni(OH)_2_ nanosheets was soaked in the solution and left at room temperature for 24 h. The nickel foam was carefully removed after the process and rinsed thoroughly with deionized water and absolute ethanol. The prepared samples were named FeNi-PBA@Ni(OH)_2_/Ni.

### 2.4. Preparation of FeNi@NiA

The FeNi-PBA@Ni(OH)_2_/Ni was electrochemically stimulated in a three-electrode cell, where a carbon rod acted as the counter electrode, mercury oxide was the reference electrode, and the FeNi-PBA@Ni(OH)_2_/Ni composite served as the working electrode. It was electrochemically activated at a constant potential of 0.65 V for 60 min. The prepared sample was named Fe-doped NiOOH@Ni(OH)_2_ (FeNi@NiA).

### 2.5. Electrochemical Characterizations

Electrochemical measurements are performed with a Zennium E electrochemistry workstation (Zahner, Germany) in a standard three-electrode system. Detailed steps can be found in the Supplementary Information (SI).

### 2.6. Structural Characterization

The phase compositions of the catalysts were characterized by X-ray diffraction (XRD) on a Rigaku Smartlab diffractometer with CuKa irradiation. The surface was examined through scanning electron microscopy (SEM) on a JEOL JSM-7001F system and energy dispersive spectroscopy (EDS) mapping was examined at a voltage of 15 kV. X-ray photoelectron spectroscopy (XPS) measurements were conducted on a Thermo Fisher scientific (Waltham, MA, USA) Escalab 250Xi; the C1s peak at 284.8 eV was taken as a reference. The microstructures, morphology, and element analysis were carried out by transmission electron microscopy (TEM) on a JEOL JEM-2001F electron microscope at an accelerating voltage of 200 kV.

## 3. Results and Discussion

**Figure 1:**
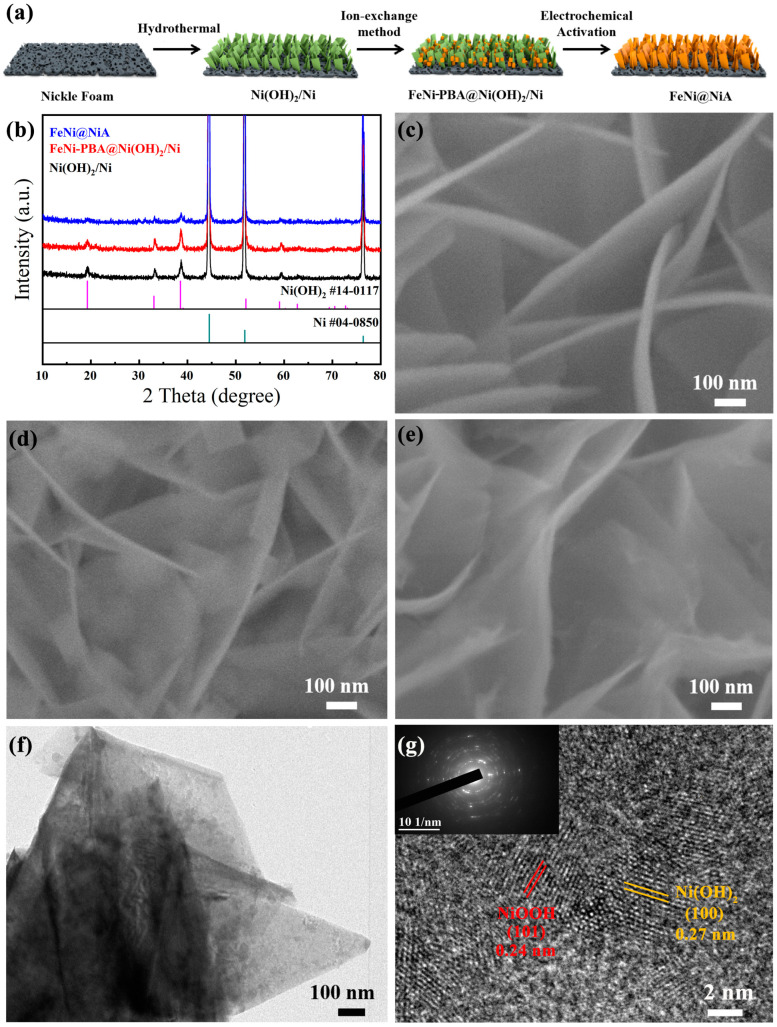
(**a**) Schematic illustration of the fabrication process of the hybrid; (**b**) XRD patterns before and after electrochemical activation; (**c**–**e**) SEM images of Ni(OH)_2_/Ni, FeNi-PBA@Ni(OH)_2_/Ni, and FeNi@NiA; (**f**) TEM image of FeNi@NiA; and (**g**) HRTEM image of FeNi@NiA (inset is the SAED pattern).

The synthesis process of FeNi@NiA is shown in Figure 1a, which mainly includes three steps. First, Ni(OH)_2_ was synthesized via a one-step hydrothermal strategy on Ni foam to serve as an Ni precursor for FeNi-PBA growth. A three-dimensional (3D) structure of Ni foam with open channels and superior electrical conductivity ensures the adequate contact of the electrodes and interfaces and accelerates the charge and gas transport. Then, the eNi(OH)_2_/Ni array was soaked in K_3_[Fe(CN)_6_] solution via a gentle ion-exchange method to obtain the bimetallic Fe-Ni PBA nanocubes supported on Ni(OH)_2_. At the boundary between solid and liquid phases, the released Ni^2+^ would coordinate with Fe(CN)_6_^3−^ in situ, then OH^−^ and Fe(CN)_6_^3−^ undergo an ion exchange, resulting in the formation of PBA nanocubes on the Ni(OH)_2_ nanosheet platforms. Finally, the FeNi@NiA electrode was obtained via electrochemical activation at 0.65 V for 1 h. In the process, the PBAs were oxidatively dissolved and transformed into the Fe-doped NiOOH, producing the resultant FeNi@NiA heterostructure.

XRD was carried out to verify the formation process of FeNi@NiA, as shown in Figure 1b. The peaks located at 44.5, 51.8, and 76.4° are associated with the (111), (200), and (220) lattice planes of FCC Ni (JCPDS NO. 04-0850). And a series of typical diffraction peaks of the XRD pattern of the nickel foam after hydrothermal treatment are well-indexed to the facets of Ni(OH)_2_ (JCPDS NO. 14-0117), illustrating the formation of Ni(OH)_2_ on nickel foam. As the ion exchange time is extended in K_3_[Fe(CN)_6_], the new diffraction peaks at 17.3, 24.6, and 35.1° can be indexed well to the (200), (220), and (400) crystal facets of KNiFe(CN)_6_ (JCPDS NO. 51-1897) (Figure S1). We noticed the diffraction peaks of FeNi-PBA become stronger with the prolongation of immersion time, indicating the growth of FeNi-PBA on the Ni(OH)_2_/NF. Notably, the diffraction peaks of FeNi-PBA disappear in the local enlarged XRD pattern of FeNi@NiA after the electrochemical activation (Figure S2). It is shown that the crystal structure of PBAs has been changed after electrochemical activation. A distinctive diffraction peak for Ni(OH)_2_ is observed in the product, when contrasted with the pre-activated one. Nevertheless, the intensity of the diffraction peak after electrochemical activation is significantly reduced, indicating the poor crystallinity of Ni(OH)_2_ after activation.

SEM was conducted to observe the morphological and structural evolution of the FeNi@NiA. As shown in Figure 1c, the Ni(OH)_2_ nanosheets are vertically aligned on Ni foam, showing a hierarchical array structure. The Ni(OH)_2_ nanosheets display a sleek, uniform surface and possess a thickness of roughly 30 nm. Such intercross and hierarchical nanosheet arrays on Ni foam are favorable for mass transfer and improving mechanical stability. The ion exchange gives rise to the FeNi-PBA nanocubes with dimensions of about 100 nm uniformly decorated on the Ni(OH)_2_ nanosheets (Figure 1c). Moreover, with the prolongation of immersion time, closely packed PBA nanocubes were grown on the Ni(OH)_2_ nanosheets (Figure S3). After the electrochemical activation in 1.0 M KOH, all the nanocubes on the Ni(OH)_2_ nanosheets (FeNi-PBA@Ni(OH)_2_/Ni) disappeared. The morphology of the nanosheets becomes irregular, indicating the in-situ formation of FeNi@NiA crumpled nanosheets with a larger geometric area to make contact more effectively with the electrolyte (Figure 1e).

TEM was performed to characterize the microstructure of the FeNi-PBA@Ni(OH)_2_/Ni (Figure S4). The PBA nanocubes are uniformly distributed on the nanosheets. The average size of FeNi-PBA nanocubes is about 100 nm. Elemental mapping confirms the evenly dispersed of Ni, O, and N in the entire nanosheets, while Fe was selectively distributed in the corresponding nanocube regions (Figure S5). After CV conditioning, the FeNi@NiA exhibits nanosheet-like structures without nanocubes on the surfaces (Figure 1f). The interspacing of the measured inter-fringe distances are in agreement with the d-spacing of (101) crystal planes of NiOOH and (100) crystal planes of Ni(OH)_2_, indicating the formation of heterostructures (Figure 1g) [36]. The elemental mapping analysis of FeNi@NiA by STEM measurement shows that the Fe, Ni, N, and O elements are homogeneously distributed on the nanosheets (Figure S6), confirming, indeed, the in-situ doping of Fe into the NiOOH@Ni(OH)_2_. The selected-area electron diffraction (SAED) pattern of the Ni(OH)_2_ nanosheet shows bright diffraction spots distributed in a regular hexagonal shape, indicating the single crystal structure exposed with a (001) facet (Figure S7) [37,38,39]. In the process of the in-situ ion exchange, nanocubes are gradually formed on nanosheets, in which monocrystals evolve into FeNi-PBA@Ni(OH)_2_/Ni of the polycrystals as shown in Figure S8. After performing CV testing, the SAED pattern of FeNi@NiA still shows the ring features (Figure 1g, inset), suggesting the preserved polycrystalline structure. Through ion exchange and electrochemical activation, the structure was changed from single-crystal to polycrystalline, which greatly increases the electrochemically active area. Such a plentiful and staggered distribution of NiOOH and Ni(OH)_2_ generates numerous interfaces and active sites, which can modulate the overall electronic energy state and thus enhance the catalytic activities [32].

**Figure 2:**
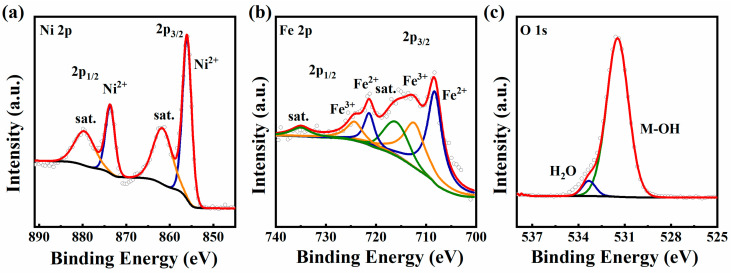
XPS spectra of FeNi@NiA: (**a**) Ni 2p; (**b**) Fe 2p; and (**c**) O 1s high-resolution XPS spectra.

The chemical composition and electronic configuration of FeNi-PBA@Ni(OH)_2_/Ni and FeNi@NiA were further evaluated by XPS. For the Ni 2p of FeNi-PBA@Ni(OH)_2_/Ni (Figure S9a), the Ni 2p XPS spectrum reveals two major peaks with binding energies at 873.7 and 856.1 eV, assigned to Ni 2p^1/2^ and Ni 2p^3/2^ [40,41]. The first doublet at 708.3 and 721.3 eV corresponds to the binding energies of Fe 2p^3/2^ and Fe 2p^1/2^ (Figure S9b), indicating the presence of Fe^2+^ in FeNi-PBA@Ni(OH)_2_/Ni. The peaks observed at 712.3 and 724.3 eV are attributable to the presence of Fe^3+^ [42]. The O 1s spectrum shows two main peaks at 531.5 and 533.4 eV (Figure S9c), corresponding to the Ni-OH from the Ni(OH)_2_ and adsorbed water species, respectively [42,43]. The peaks at 854.84 and 872.34 eV can be assigned to the Ni^2+^ of FeNi@NiA, while the peaks at 856.49 and 874.05 eV are related to the Ni^3+^ of the FeNi@NiA (Figure 2a), which provide solid evidence for the formation of NiOOH [44,45]. The first doublet at 708.3 and 721.9 eV corresponds to the binding energies of Fe 2p^3/2^ and Fe 2p^1/2^ (Figure 2b). The second one at 712.6 and 725.8 eV can be assigned to Fe^3+^, suggesting the binding energy of Fe is slightly positively shifted after electrochemical activation. For the O 1s spectrum, the peaks located at 531.5 and 533.4 eV are ascribed to the M-OH and the adsorbed water, respectively (Figure 2c). Moreover, the peaks at 530.5 eV are ascribed to the Ni-O-Ni bond in the O 1s spectrum of FeNi@NiA, which further prove the constitution of the NiOOH after electrochemical activation (Figure 2c) [42,45]. The above results indicate the successful construction of Fe-doping NiOOH/Ni(OH)_2_ heterostructures, which will effectively regulate the electronic energy state of the active sites in catalytic reactions. This modulation impacts the Gibbs free energy and the dynamics of charge transfer, thereby promoting the catalyst’s performance [46].

**Figure 3:**
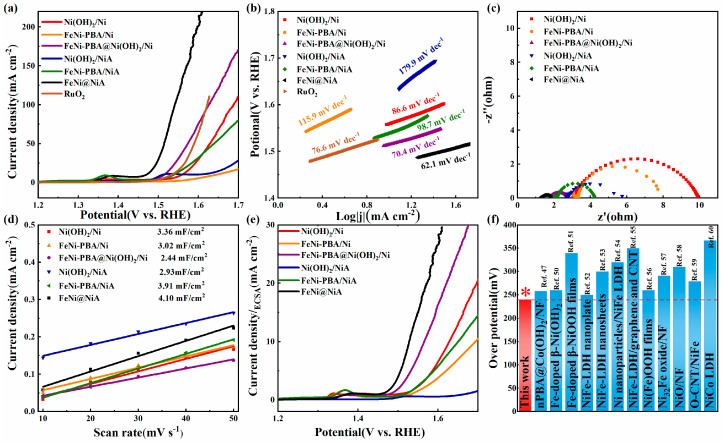
(**a**) The polarization curves at a scan rate of 5 mV/s (with iR correction) of various electrocatalysts for OER in 1.0 M KOH solution; (**b**) Tafel plots; (**c**) EIS spectrum (at a bias voltage of 1.5 V vs. RHE with the frequency range from 0.1 Hz to 100 K Hz) and (**d**) C_dl_ of various electrocatalysts determined from the CV curves measured across a spectrum of scan rates; (**e**) polarization curves with current density normalized to the ECSA; and (**f**) comparison of the overpotential of FeNi@NiA at 10 mA/cm^2^ in this study with those of other catalysts documented in recent publications.

The catalytic performances of the FeNi@NiA nanosheets for OER were investigated through linear sweep voltammetry conducted in an N_2_-saturated 1.0 M KOH in a three-electrode system. An iR compensation was implemented for all the initial electrochemical data. As shown in Figure 3a, linear sweep voltammetry (LSV) curves reveal that the FeNi@NiA electrode presents the lowest overpotential of 240 mV at a current density of 10 mA/cm^2^, which is much smaller than those of FeNi-PBA@Ni(OH)_2_/Ni (285 mV), FeNi-PBA/NiA (310 mV), FeNi-PBA/Ni (415 mV), Ni(OH)_2_/Ni (358 mV), Ni(OH)_2_/NiA (370 mV), and RuO_2_ loaded on Ni foam (303 mV). The much lower overpotential of the FeNi@NiA indicates remarkable OER activity under alkaline conditions. To further assess the intrinsic catalytic activity, the kinetics of OER were evaluated through the corresponding Tafel plots (Figure 3b). The Tafel slope has been used to dissect the kinetics of the rate-determining step in the OER process. The Tafel slope of the FeNi@NiA is only 62.1 mV dec^−1^, which is vastly superior to those of FeNi-PBA@Ni(OH)_2_/Ni (70.4 mV dec^−1^), FeNi−PBA/NiA (98.7 mV dec^−1^), FeNi-PBA/Ni (115.9 mV dec^−1^), Ni(OH)_2_/NiA (179.9 mV dec^−1^), Ni(OH)_2_/Ni (86.6 mV dec^−1^), and RuO_2_ (76.6 mV dec^−1^). The lowest Tafel slope of FeNi@NiA reveals the fastest OER kinetics and the easiest charge transfer process. Electrochemical impedance spectroscopy (EIS) analysis was performed on all the samples (Figure 3c). The FeNi@NiA shows the smallest charge-transfer resistance (R_ct_), which is considerably smaller than all the other values, respectively. This indicates that faster electron transfer and ion transport occur at the FeNi@NiA interface. Additionally, the double-layer capacitance (*C*_dl_), which is generally used to estimate the electrochemical surface area (ECSA), was measured to assess the catalytic performance of the FeNi@NiA electrode (Figure 3d). The FeNi@NiA electrode has the highest *C*_dl_ value (4.10 mF/cm^2^), which is much higher than that of NiFe-PBA@Ni(OH)_2_/Ni and Ni(OH)_2_/Ni, revealing that the construction of the Fe-NiOOH@Ni(OH)_2_ heterojunction and the large specific surface area of Ni(OH)_2_ nanosheets endow the material with more OER active sites and can provide more electron pathways. FeNi@NiA exhibited the largest ECSA value of 102.5 (Table S1), indicating the most exposed active sites, thus promoting increased activity. To evaluate the intrinsic activity of the specific active site, we normalized the OER current to the ECSA (*j*_ECSA_) (Figure 3e). FeNi@NiA shows a *j*_ECSA_ of 10 mA/cm^2^ at a low overpotential of 297 mV. At the same overpotential, NiFe-PBA@Ni(OH)_2_/NF and Ni(OH)_2_/NF, respectively, exhibit a much smaller *j*_ECSA_ of 4.42 and 1.36 mA/cm^2^, and the bare Ni foam has the smallest *j*_ECSA_ of only 1.2 mA/cm^2^. These results also indicate that the intrinsic activity of FeNi@NiA is much higher than that of FeNi-PBA@Ni(OH)_2_/Ni and Ni(OH)_2_/Ni [47].

The synergistic effect of the FeNi@NiA heterostructure not only increased the highly electrochemically active surface area but also can further facilitate its intrinsic activity for OER through modulating the overall electronic energy state. On this basis, the incorporation of Fe can promote the conductivity of NiOOH and optimize the adsorption energy of OER intermediates [48,49]. Most significantly, the results also confirm that the outstanding OER activity of Fe-NiOOH@Ni(OH)_2_ is also attributed to the increased ECSA and the enhanced intrinsic activity of each active site in converting OH^−^ into O_2_. The FeNi@NiA demonstrates the most minimal overpotential of 240 mV at *j*_geo_ = 10 mA/cm^2^, which surpasses many other state-of-the-art OER electrocatalysts (Figure 3f) [47,50,51,52,53,54,55,56,57,58,59,60].

**Figure 4:**
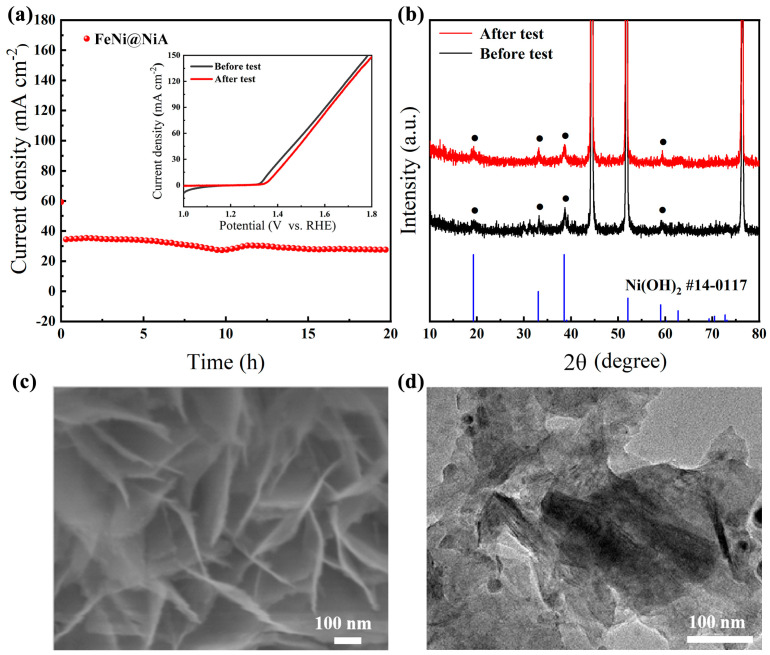
(**a**) The chronoamperometric measurement of FeNi@NiA for OER at an overpotential of 0.7 V (inset is the LSV curves of before and after durability test); (**b**) XRD pattern; (**c**) SEM image; and (**d**) TEM image of FeNi@NiA after durability test.

The stability of the catalyst is also vital to its commercial application. Long-term durability measurements were carried out by chronoamperometric and cyclic voltammetry in 1 M KOH of FeNi@NiA. The current density remained stable without significant degradation over 20 h (Figure 4a). The polarization curve exhibited minimal change in comparison to its initial state (Figure 4a, inset), indicating that the sample still maintains high OER activity and excellent durability due to the robust heterostructures. The oxyhydroxide and hydroxide can improve the durability of the electrocatalyst in a strong alkaline environment. After the electrochemical test, the collected sample shows similar XRD patterns as the initial sample, suggesting that the phase structure nearly does not change. The SEM and the TEM characterization also verify the well-retained microstructure of FeNi@NiA. The lattice finger of 0.27 nm in the HRTEM image can be still found and ascribed to the lattice plane (100) of Ni(OH)_2_ (Figure S10), suggesting that the crystal structure of FeNi@NiA remains unchanged even after a long-term durability test.

**Figure 5:**
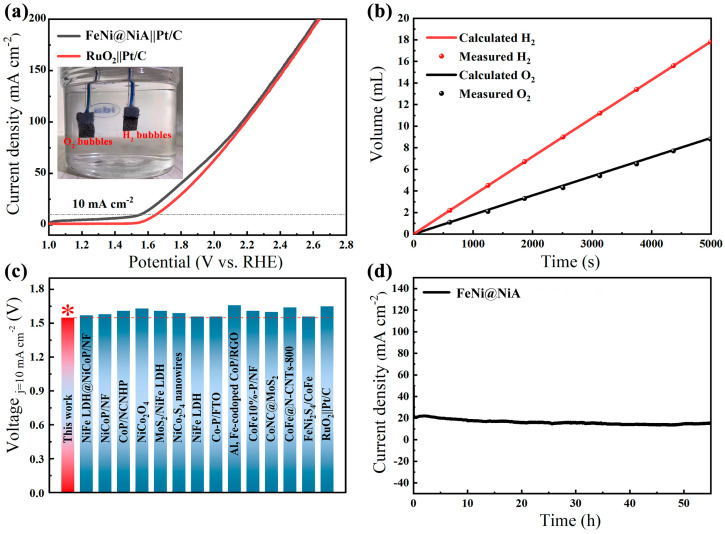
(**a**) LSV of the typical two-electrode system using FeNi@NiA as the anode and Pt/C/Ni as the cathode in 1.0 M KOH; commercial RuO_2_/Ni||Pt/C/Ni was also tested for comparison (inset: photograph of the two-electrode device); (**b**) the volumes of H_2_ and O_2_ gases generated in relation to the duration of the water-splitting process; (**c**) the comparison of cell voltages for achieving a 10 mA cm^−2^ for the FeNi@NiA with the performance of other OER catalysts from recent reports; and (**d**) time-dependent current density curve for FeNi@NiA in a two-electrode cell.

We further evaluated the overall water-splitting performance involved in assigning the FeNi@NiA electrode as the anode and the commercial Pt/C/Ni as the cathode in a two-electrode system. As shown in Figure 5a, the cell delivers a current density of 10 mA/cm^2^ at a voltage of 1.55 V. This value is much lower than that of an electrolyzer constructed by an RuO_2_/Ni anode and a Pt/C/Ni cathode (1.65 V). As a proof of demonstration, the electrolyzer can be driven using a 1.5 V commercial dry battery as the energy source (inset in Figure 5a). Notably, the performance at the current density of 10 mA/cm^2^ also surpasses most non-noble metal catalysts for overall alkaline water splitting (Figure 5c) [61,62,63,64,65,66,67,68,69,70,71,72,73]. The cathode/anode exhibits steady rates of H_2_ and O_2_ evolution with a ratio of approximately 2:1, which matches well with the theoretical value during electrolysis and indicates an almost perfect 100% Faradaic efficiency (Figure 5b). This proves that FeNi@NiA is an efficient and durable electrocatalyst for overall water splitting. The durability test of FeNi@NiA shows a stable potential at 20 mA/cm^2^ for over 55 h, both suggesting stability for water splitting (Figure 5d). The continuous generation of gas bubbles are visible on the electrode surfaces during the electrolysis process.

## 4. Conclusions

In summary, we achieved the in-situ construction of Fe-doped NiOOH on hierarchical Ni(OH)_2_ nanosheet arrays as a flexible and precise treatment method on nickel foam acting as a state-of-the-art electrode. Electrochemical activation was applied to synthesize in-situ surface-activated FeNi@NiA self-supporting electrodes with the nanosheet structure from the templated NiFe-based PBA nanocubes. The formation of the FeNi@NiA heterojunction is demonstrated to be more thermodynamically favorable for OER in alkaline electrolytes, which exhibited an impressive overpotential of 240 mV to achieve a current density of 10 mA/cm^2^. As explored for overall water splitting, the alkali-electrolyzer based on the FeNi@Ni achieves a current density of 10 mA/cm² with a minimal cell voltage of 1.55 V with outstanding durability over 55 h. This work opens up a new avenue for the reconstruction of Prussian blue analogues, while also presenting a straightforward approach to crafting potent, economical, and efficient OER electrocatalysts.

## Data Availability

All data generated for this work are included in this article.

## Supplementary Materials

The following supporting information can be downloaded at: https://www.mdpi.com/article/10.3390/ma17184670/s1, Figure S1: XRD pattern for FeNi-PBA@Ni(OH)_2_/Ni with different immersion time of 0 h, 2 h, 12 h, 24 h, and 48 h; Figure S2: XRD pattern for FeNi@NiA and FeNi-PBA@Ni(OH)_2_/Ni; Figure S3: SEM pattern for FeNi-PBA@Ni(OH)_2_/Ni with different immersion time of 2 h, 12 h, 24 h, and 48 h; Figure S4: The low-resolution TEM images of FeNi-PBA@Ni(OH)_2_/Ni; Figure S5: High-angle annular dark field-TEM image of FeNi-PBA@Ni(OH)_2_/Ni and the corresponding elemental mapping images of Fe, Ni, N, and O; Figure S6: High-angle annular dark field-TEM image of FeNi@NiA and the corresponding elemental mapping images of Fe, Ni, N, and O; Figure S7: SAED pattern of Ni(OH)_2_/Ni; Figure S8: SAED pattern of FeNi-PBA@Ni(OH)_2_/Ni; Figure S9: XPS of FeNi-PBA@Ni(OH)_2_/Ni (a) Ni 2p, (b) Fe 2p, and (c) O 1s; Figure S10: HR-TEM image of FeNi@NiA after durability test; Table S1: The ECSA of the sample.
