# Supplementary material for: In-Situ Construction of Fe-Doped NiOOH on the 3D Ni(OH)2 Hierarchical Nanosheet Array for Efficient Electrocatalytic Oxygen Evolution Reaction
Source: Materials (Basel). 2024 Sep 23;17(18):4670. doi: 10.3390/ma17184670 (PMC11434255; doi:10.3390/ma17184670)
Supplement: Supplementary file 1 [file materials-17-04670-s001.zip › materials-3155104-supplementary.pdf]

Supporting information

In-Situ Construction of Fe-Doped NiOOH on the 3D $Ni(OH)_2$ Hierarchical Nanosheet Array for Efficient Electrocatalytic Oxygen Evolution Reaction

Preparation of $Ni(OH)_2$/Ni

$Ni(NO_3)_2 \cdot 6H_2O$ (0.131g), urea (0.225 g), NH_4F (0.056g) were dissolved in 150 mL of water and magnetically stirred until completely dissolved. The size of Ni foam was 1.5 cm*4cm with a thickness of 1.5 mm. It was cleaned by acetone and ethanol under ultrasonication conditions for 10 min, respectively and then rinsed repeatedly with ethanol and water. The pretreated Ni foam was immersed into the solution and the solution was transferred to a stainless Teflon-lined autoclave with the inner volume of 100 mL and placed in a drying oven at a temperature of 120 °C for 2h. When the temperature naturally cooled down to room temperature, the as-obtained Ni foam with catalysts on it was washed with water and ethanol for several times to remove the residual ingredients. The prepared sample was named as $Ni(OH)_2$/NF.

Preparation of FeNi-PBA@$Ni(OH)_2$/Ni

$K_3[Fe(CN)_6]$ (0.033 g) was dissolved in 20 mL of water and magnetically stirred until completely dissolved. The nickel foam with $Ni(OH)_2$ nanosheets was soaked in the solution and left at room temperature for 24 h. After completion, the nickel foam was taken out and rinsed several times with deionized water and absolute ethanol. The prepared samples were named FeNi-PBA@$Ni(OH)_2$/Ni.

Preparation of FeNi@NiA

The FeNi-PBA@$Ni(OH)_2$/Ni were electrochemically activated. In the three-electrode system, a carbon rod was used as the counter electrode, mercury oxide was used as the reference electrode, and the as-prepared FeNi-PBA@$Ni(OH)_2$/Ni was used as the working electrode. It was electrochemically activated at a constant potential of 0.65 V for 60 min. The prepared sample was named as Fe-doped NiOOH@$Ni(OH)_2$ (FeNi@NiA).

Electrochemical characterizations

Electrochemical measurements are performed with a Zennium E electrochemistry workstation (Zahner, Germany) in a standard three-electrode system. FeNi@NiA, a graph-ite carbon rod and a mercury oxide electrode (Hg/HgO) served as working electrode, counter electrode, and reference electrode, respectively. Potentials are reported versus the reversible hydrogen electrode (RHE) via the following equation: $E_{RHE} = E_{Hg/HgO} + 0.059$ pH + 0.098 V. Polarization curves measurements were conducted in N_2 saturated 1.0 M KOH solution with a scan rate of 5 mV s^{-1}. Double layer capacitance measurements were con-ducted by varying the scan rates (10-50 mV s^{-1} with an interval 10 mV) in a potential window nearly without Faradaic process. The polarization curves were established as overpotential vs log current (log j) to get Tafel plots for evaluating the OER reaction kinet-ics of obtained catalysts. By fitting the Tafel plots (the linear portion) to the Tafel equation ($\eta = b \log(j) + a$), the Tafel slope can be obtained. The Electrochemical impedance spec-troscopy (EIS) measurements for the FeNi@NiA were performed at a bias voltage of 1.5 V vs. RHE with the frequency range from 0.1 Hz to 100 K Hz. Long-term stability was measured by chronoamperometry at the current density of 20 mA cm^{-2} over 20 hours. For RuO_2, 5 mg of the sample and 5 μL 5% Nafion solution were put in 800 μL mixture of water/ethanol with a volume

ratio of 3:1 and dispersed by ultrasonication for 30 min to form a homogeneous ink. After that, 20 μL of this ink was carefully dropped onto a Ni foam with an area of 1*1 cm^2 for 5 times and dried in air.

Overall water splitting was measured in a two-electrode system. Fe-doped NiOOH@$Ni(OH)_2$ worked as the anode and commercial Pt/C supported on NF as the cathode in 1.0 M KOH electrolyte with a scan rate of 5 mV s^{-1} over a potential range of 1 to 3 V. The volumes of H_2 and O_2 produced were collected at 3 V to calculate the Faradaic Efficiency with the following equation:

$$FE\ (\%) = nFV_m/QV_m*100\%$$

Where n represents the reactive electron number, F represents the Faraday constant with a value of 96,485 C mol^{-1}, Q with a value of the total charge passed through the elec-trodes, V_m represents the molar volume of gas. The durability test of FeNi@NiA showed a stable potential at 20 mA cm^{-2} for over 55 h.

ECSA=C_{dl}/C_s, where C_{dl} is the measured double-layer capacitance, and C_s is the capacitance value.

Structural Characterization

The phase compositions of the catalysts were characterized by X-ray diffraction (XRD) on a Rigaku Smartlab diffractometer. The surface and elemental distribution were exam-ined through scanning electron microscopy (SEM) and energy dispersive spectroscopy (EDS) mapping on a JEOL JSM-7001F system. X-Ray photoelectron spectroscopy (XPS) measurements were conducted on a Thermo fisher scientific Escalab 250Xi. The micro-structures, morphology and element analysis were carried out by transmission electron microscopy (TEM) on a JEOL JEM-2001F electron microscope.

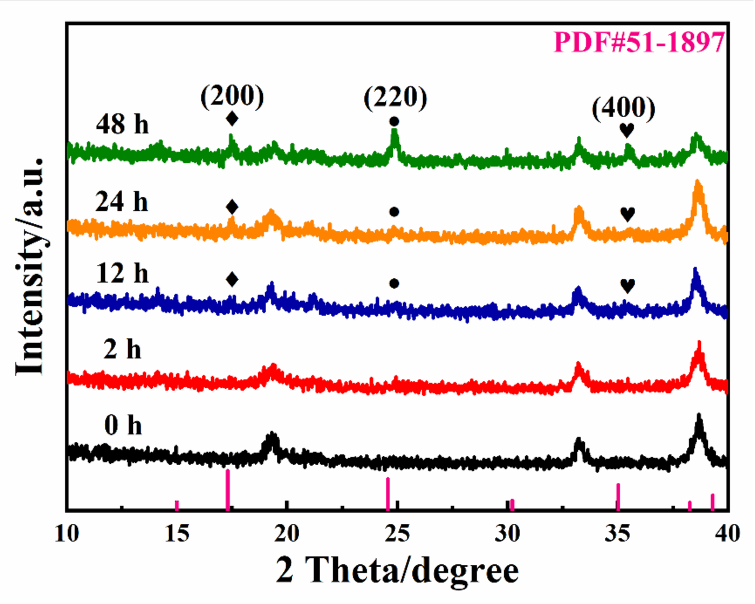

Figure S1: XRD pattern for FeNi-PBA@$Ni(OH)_2$/Ni with different immersion time of 0h, 2h, 12h, 24h and 48h.

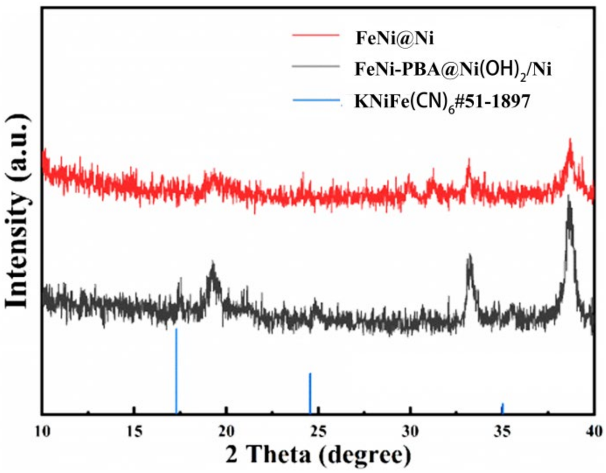

Figure S2: XRD pattern for FeNi@NiA and FeNi-PBA@Ni(OH)$_2$/Ni.

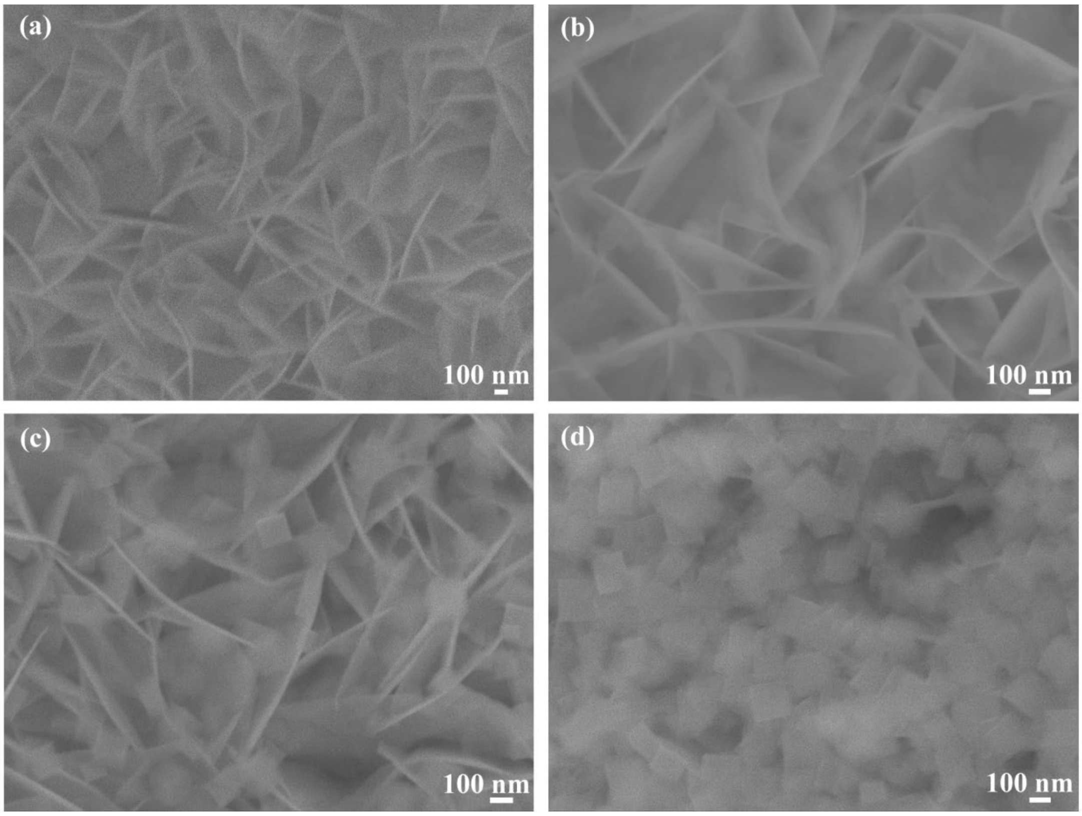

Figure S3: SEM pattern for FeNi-PBA@$Ni(OH)_2$/Ni with different immersion time of 2h, 12h, 24h and 48h.

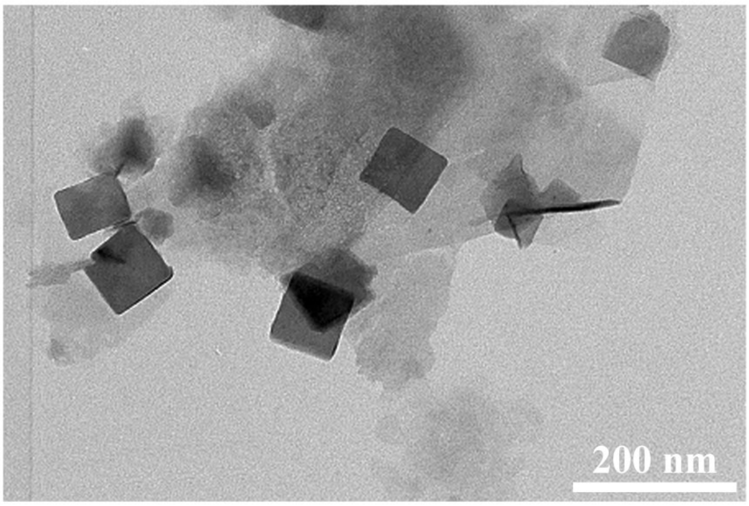

Figure S4: The Low-resolution TEM images of FeNi-PBA@$Ni(OH)_2$/Ni.

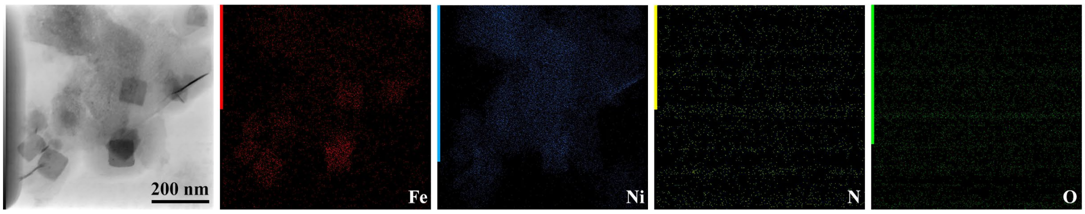

Figure S5: High-angle annular dark field-TEM image of FeNi-PBA@Ni(OH)$_2$/Ni and the corresponding elemental mapping images of Fe, Ni, N and O.

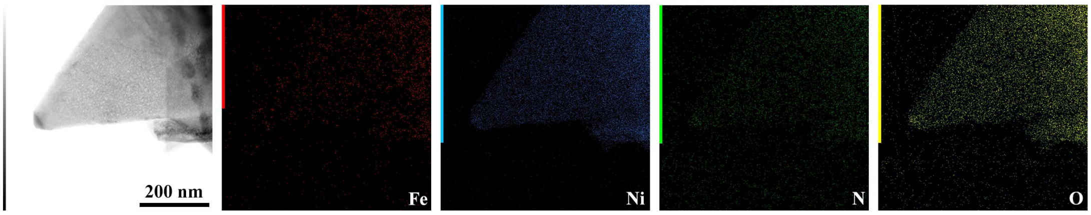

Figure S6: High-angle annular dark field-TEM image of FeNi@NiA and the corresponding elemental mapping images of Fe, Ni, N and O.

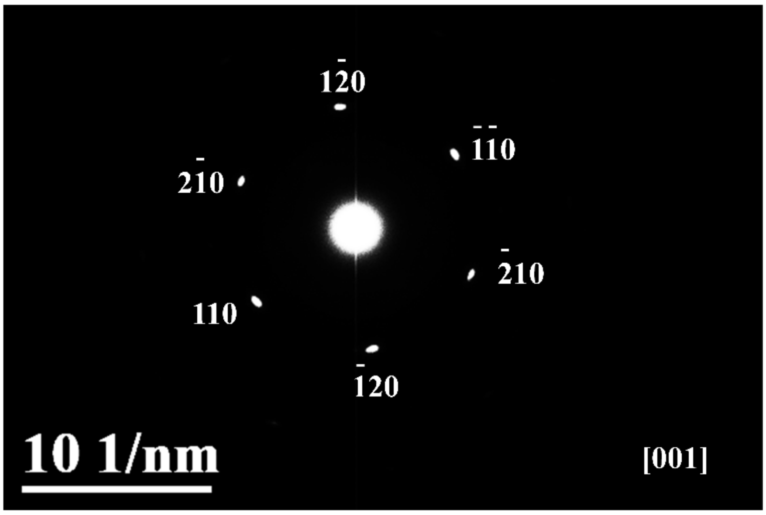

Figure S7: SAED pattern of $Ni(OH)_2/Ni$.

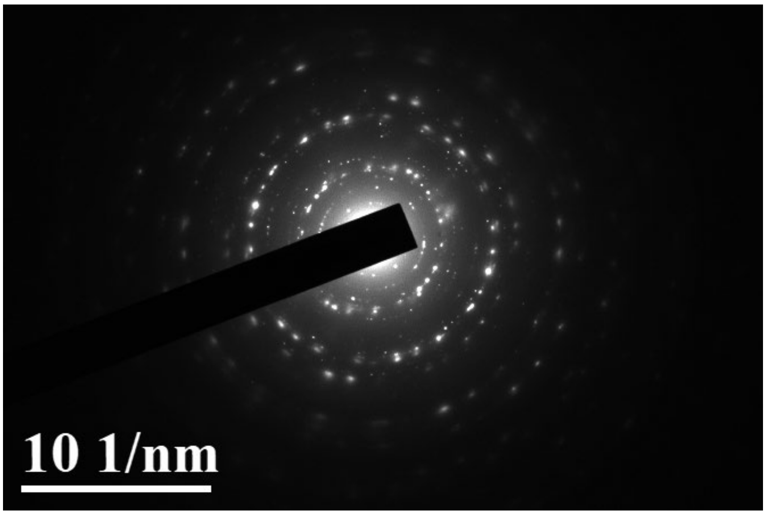

Figure S8: SAED pattern of FeNi-PBA@Ni(OH)2/Ni.

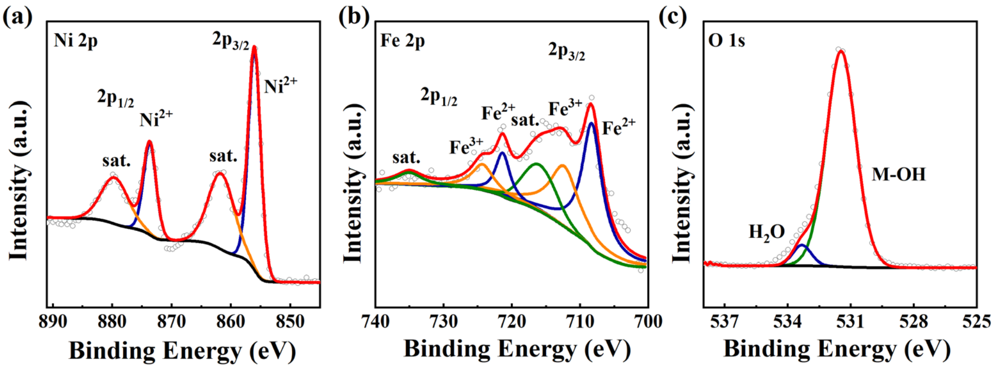

Figure S9: XPS of FeNi-PBA@Ni(OH)2/Ni (a) Ni 2p, (b) Fe 2p, (c) O 1s.

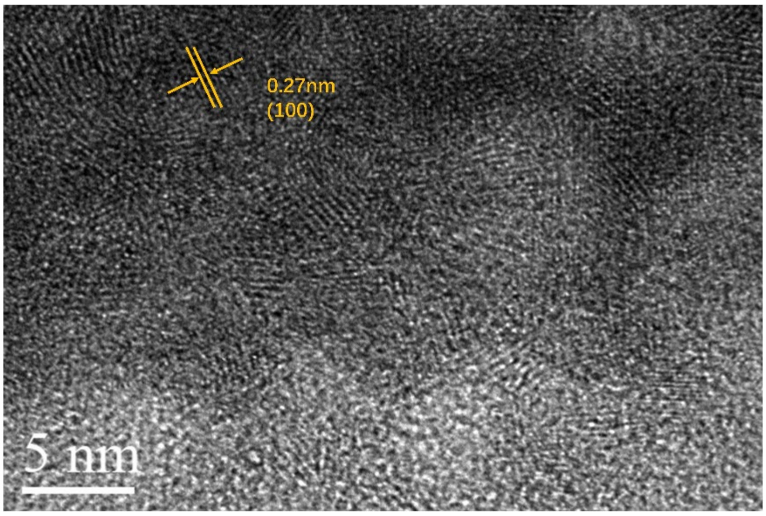

Figure S10: HR-TEM image of FeNi@NiA after durability test.

Table S1 The ECSA of the samples

Sample	$Ni(OH)_2$/Ni	FeNi-PBA/Ni	FeNi-PBA@$Ni(OH)_2$/Ni	$Ni(OH)_2$/NiA	FeNi-PBA/NiA	FeNi@NiA
ECSA	84	75.5	61	73.25	97.75	102.5
